# Interaction of Cholesterol With the Human SLC1A5 (ASCT2): Insights Into Structure/Function Relationships

**DOI:** 10.3389/fmolb.2019.00110

**Published:** 2019-10-23

**Authors:** Mariafrancesca Scalise, Lorena Pochini, Jessica Cosco, Emma Aloe, Tiziano Mazza, Lara Console, Antonella Esposito, Cesare Indiveri

**Affiliations:** Unit of Biochemistry and Molecular Biotechnology, Department DiBEST (Biologia, Ecologia, Scienze della Terra), University of Calabria, Cosenza, Italy

**Keywords:** membrane transport, proteoliposomes, SLC, cholesterol, chemical targeting, mercury, cysteine, tryptophan

## Abstract

The human SLC1A5 commonly known as ASCT2 is a sodium-dependent neutral amino acid antiporter involved in transmembrane traffic of glutamine that is exchanged through the cell membrane with smaller amino acids such as serine or threonine. Due to the strong overexpression in human cancers, ASCT2 is widely studied for its relevance to human health. Of special interest are the aspects related to the regulation of its function. The role of cholesterol as a modulator of the transport activity has been studied using a combined strategy of computational and experimental approaches. The effect of cholesterol on the ${\text{Na}}_{\text{ex}}^{+}$-[^3^H]glutamine_ex_/glutamine_in_ antiport in proteoliposomes has been evaluated by adding cholesteryl hemisuccinate. A strong stimulation of transport activity was observed in the presence of 75 μg cholesteryl hemisuccinate per mg total lipids. The presence of cholesterol did not influence the proteoliposome volume, in a wide range of tested concentration, excluding that the stimulation could be due to effects on the vesicles. cholesteryl hemisuccinate, indeed, improved the incorporation of the protein into the phospholipid bilayer to some extent and increased about three times the V_max_ of transport without affecting the K_m_ for glutamine. Docking of cholesterol into the hASCT2 trimer was performed. Six poses were obtained some of which overlapped the hypothetical cholesterol molecules observed in the available 3D structures. Additional poses were docked close to CARC/CRAC motifs (Cholesterol Recognition/interaction Amino acid Consensus sequence). To test the direct binding of cholesterol to the protein, a strategy based on the specific targeting of tryptophan and cysteine residues located in the neighborhood of cholesterol poses was employed. On the one hand, cholesterol binding was impaired by modification of tryptophan residues by the Koshland's reagent. On the other hand, the presence of cholesterol impaired the interaction of thiol reagents with the protein. Altogether, these results confirmed that cholesterol molecules interacted with the protein in correspondence of the poses predicted by the docking analysis.

## Introduction

The human ASCT2 transporter is one of the seven members of the SLC1 family (SLC1A5) and represents one of the most studied proteins among the SLC members being considered a hot spot topic for either biochemical interest and pharmacological applications (Kanai et al., 2013; Scalise et al., 2019). ASCT2 shows a broad tissue expression. The first pioneering studies on the mice and human isoforms, conducted in intact cells, showed that this transporter has a preference toward neutral amino acids (Kekuda et al., 1996; Utsunomiya-Tate et al., 1996; Torres-Zamorano et al., 1998; McGivan and Bungard, 2007) and displays a peculiar transport mode: an antiport of neutral amino acids coupled to co-transport of Na^+^ from the external to the internal side of the cell membrane. Soon after, the rat transporter (Oppedisano et al., 2004, 2007) was studied employing the proteoliposome technology that allows investigating a single protein inserted in an artificial membrane with the same orientation as in the native cell membrane (Scalise et al., 2017a). These studies confirmed most of the characteristics described in intact cells. The human protein has been then obtained by overexpression in *Pichia pastoris* and purification by affinity chromatography (Pingitore et al., 2013). Novel features of the human ASCT2 have been revealed thanks to the proteoliposome tool (Scalise et al., 2014). Functional asymmetry of the transporter has been described: the ASCT2 revealed to be competent for the bidirectional transport of glutamine, asparagine, threonine, and serine while alanine can be only inwardly transported. Kinetic asymmetry has been also demonstrated with external affinities toward substrates in the micromolar range and internal affinities in the millimolar range. These parameters correlate with the extra and intracellular concentrations of the amino acids (Cynober, 2002; Pingitore et al., 2013; Scalise et al., 2014). Interestingly, cysteine, i.e., one of the amino acids underlying the acronym ASC(Cysteine)T2, has been shown to be a modulator of the transporter but not a substrate (Scalise et al., 2015) explaining overlooked old data (Utsunomiya-Tate et al., 1996). This peculiar regulation mode, together with the discovered responsiveness to GSH, H_2_S, and NO suggested that ASCT2 could be a redox sensor in physiological and pathological conditions. This was confirmed by site-directed mutagenesis identifying key residues for the redox sensing (Scalise et al., 2018b). An interesting and controversial aspect is the electrical nature of the transport reaction that has been solved in the proteoliposome model by specifically setting the experimental conditions close to the physiological milieu: the ASCT2 mediated Na^+^ dependent antiport is electrogenic involving at least one Na^+^ ion per transport cycle (Scalise et al., 2014). Combining *in vivo* and *in vitro* approaches, novel aspects of ASCT2 biology have been revealed. ASCT2 contains PDZ binding domain allowing for interaction with PDZK1, a well-known scaffold protein which takes contact with several plasma membrane transporters and regulates either activity and/or stability of the interactors (Dephoure et al., 2008). Furthermore, the molecular determinants for trafficking to the plasma membrane, i.e., glycosyl residues linked to asparagine 163 and 212, have been characterized. Glycosylation is required for both routing the transporter to the definitive location and for stabilizing the protein while it is not needed for intrinsic transport function (Console et al., 2015). From the findings obtained in different experimental systems, it can be deduced that the main physiological role of ASCT2 consists in mediating cell uptake of glutamine and balancing the amino acid pools in several tissues. ASCT2 has been also reported to be involved in the glutamine/glutamate cycle between astrocytes and neurons allowing for both the recycle of glutamate from the synaptic cleft in astrocytes and its re-synthesis in neurons (Broer et al., 1999; Leke and Schousboe, 2016). However, it has to be stressed that the enormous interest in ASCT2 derives from the well-acknowledged involvement in cancer development and growth. Indeed, ASCT2 is overexpressed in virtually all human cancers so far analyzed thus making this transporter a valuable target for novel drugs (Bhutia and Ganapathy, 2016; Scalise et al., 2017b; Schulte et al., 2018). Few molecules revealed to be potent inhibitors of ASCT2 and one of these, i.e., V-9302, has been tested in cell culture, tumor xenograft, and mice model for cancers (Schulte et al., 2018), even though the specificity of V-9302 is still controversial (Broer et al., 2018). The hASCT2 overexpression can find an explanation on at least two molecular events. At first, on the metabolic point of view, over-expression of hASCT2 provides cancer cells with glutamine, one of the major nutrients for cells under high proliferative state, in exchange with other amino acids such as serine deriving from glucose metabolism (Scalise et al., 2017b). At second, the glutamine taken up by hASCT2 may play also a role in cell signaling, for cell growth and development, due to the regulation of mTOR pathway with the sensing of amino acids availability in cells (Chantranupong et al., 2015; Rebsamen et al., 2015). Deciphering other regulatory properties and structure/function relationships of ASCT2 is thus of primary interest. In this respect, very recently cholesterol revealed to be important for several plasma membrane transporters function and stability (Penmatsa et al., 2013; Coleman et al., 2016; Dickens et al., 2017; Garcia et al., 2019). The presence of protein-bound cholesterol, in the form of Cholesteryl HemiSuccinate (CHEMS), has been hypothesized in the Cryo-EM structures of the hASCT2 trimer in both inward and outward-facing conformations (Garaeva et al., 2018; Yu et al., 2019). In the present study, we sought to investigate the relationships among cholesterol interacting with ASCT2 and modulation of its transport activity.

## Materials and Methods

### Materials

The *P. pastoris* wild type strain (X-33), the pPICZB vector, zeocin, Ni-NTA agarose resin were from Invitrogen; anti-rabbit IgG HRP conjugate from Cell Signaling; PD-10 columns, ECL plus, Hybond ECL membranes were from GE Healthcare; L-[^3^H]Glutamine was from Perkin Elmer; anti-ASCT2 (rabbit) was from Millipore; conjugated anti-His antibody, C_12_E_8_, Cholesteryl hemisuccinate, Amberlite XAD-4, egg yolk phospholipids (3-sn-phosphatidylcholine from egg yolk), Sephadex G-75, L-glutamine, methyl-β-cyclodextrin (MβCD) and all the other reagents were from Sigma-Aldrich.

### Recombinant Production of hASCT2 WT and Mutants

To obtain the recombinant hASCT2-6His proteins, 10 μg of pPICZB-ASCT2-6His WT or mutant constructs were linearized with PmeI and used to transform *P. pastoris* wild type strain X-33 by electroporation (Oberg et al., 2011). Putative multi-copy recombinants were selected using YPDS plates containing 2,000 μg/ml zeocin and analyzed after 3 days as previously described (Scalise et al., 2018b). For large scale protein production, transformed *P. pastoris* cells were inoculated in BMGY medium and grown at 30°C under rotatory stirring. To over-express hASCT2 WT and mutants, *P. pastoris* cells were centrifuged to remove the BMGY medium and resuspended at final OD of 1 in 250 mL BMMY medium added with 0.5% of methanol and placed in a 2 L conical flask. The growth in methanol was performed at 30°C under rotatory stirring, for 3 days adding fresh methanol every 24 h. To isolate the membrane fraction, 30 g of *P. pastoris* cells were resuspended in 300 ml of a buffer containing 50 mM Tris HCl pH 7.4, 150 mM NaCl, 6 mM β-mercaptoethanol, and 0.5 mM PMSF and disrupted using a bead beater (BioSpec Product). The chamber of the bead beater was loaded with the cell suspension mixed with glass beads (0.5 mm). After 5 min, almost 90% of the cell wall was destructed. The cell suspension was centrifuged in a JA30.50 rotor at 108,000 g for 30 min and the supernatant containing membrane and cytosolic fractions was collected. The supernatant was centrifuged in a JA30.50 rotor at 108,000 g for 90 min. The resulting membrane pellet was washed with urea buffer (5 mM Tris HCl pH 7.4, 2 mM EDTA, 2 mM EGTA, and 4 M urea) and then centrifuged as above described. The pellet containing the washed membrane fraction was resuspended at a final concentration of about 300 mg/ml in a buffer containing 25 mM Tris HCl pH 7.4, 250 mM NaCl, 6 mM β-mercaptoethanol and, 10% glycerol and homogenized using a potter homogenizer. Aliquots of 3 mL of the membrane fraction were stored at −80°C before solubilization.

### Solubilization and Purification of hASCT2 WT and Mutants

For a large-scale solubilization and purification of WT hASCT2 protein and mutants, about 1.5 g of washed membranes (400 mg/mL) were solubilized using a buffer containing 25 mM Tris HCl pH 7.4, 250 mM NaCl, 6 mM β-mercaptoethanol, 1 mM L-glutamine, 10% glycerol, and 2% C_12_E_8_ (w/w) by rotatory stirring for 3 h at 4°C. Then, centrifugation at 18,000 g for 45 min was performed and the supernatant was recovered for purification. The supernatant was applied to 2 mL Ni-nitrilotriacetic acid (NTA) agarose resin pre-equilibrated with the equilibration buffer (20 mM Tris HCl pH 7.4, 300 mM NaCl, 10% glycerol, 6 mM β-mercaptoethanol, 0.03% C_12_E_8_, 1 mM L-glutamine, and 50 mM imidazole). The resin, after supernatant application, was incubated over night with gentle agitation at 4°C to allow specific binding of recombinant hASCT2 to the NiNTA resin. After incubation, the Ni-NTA resin was packed into a column and washed with 30 mL of the equilibration buffer. Then, 10 mL of the elution buffer (20 mM Tris HCl pH 7.4, 30 0mM NaCl, 10% glycerol, 6 mM β-mercaptoethanol, 0.03% C_12_E_8_, 1 mM L-glutamine, and 500 mM imidazole) were added. Fractions containing purified protein were pooled to 2.5 mL and desalted on a PD-10 desalting column pre-equilibrated with desalting buffer (20 mM Tris HCl pH 7.4, 100 mM NaCl, 10% glycerol, 6 mM β-mercaptoethanol, 0.03% C_12_E_8_, and 1 mM L-glutamine), from which 3.5 mL were collected.

### Preparation of Cholesteryl Hemisuccinate

Cholesteryl HemiSuccinate (CHEMS) was prepared in 5% C_12_E_8_, to reach the final concentration required in the initial reconstitution mixture (see section Reconstitution of the hASCT2 WT and mutants into liposomes) and 20 mM Tris HCl pH 8.0. The solubilization was performed by two sonication cycles of 2 min (no pulse, 40 W) with a Vibracell VCX-130 sonifier as previously suggested (Hanson et al., 2008). The solubilized CHEMS was centrifuged for 5 min at 10,000 g and the supernatant was added to sonicated liposomes for 30 min under rotatory stirring (1,200 rpm) at 23°C before reconstitution.

### Protein Treatments With NEM and Koshland's Reagent

The purified WT or mutants hASCT2 were incubated with 0.5 mM NEM during the transport assay below described. In the case of the Koshland's reagent (Giangregorio et al., 2019), the purified WT hASCT2 was incubated with 0.5 mM of the reagent under rotatory stirring (1,200 rpm) at 23°C for 15 min before reconstitution.

### Reconstitution of the hASCT2 WT and Mutants Into Liposomes

The purified WT hASCT2 protein and mutants were reconstituted by removing the detergent using the batch-wise method in which mixed micelles of detergent, protein and phospholipids were incubated with 0.5 g Amberlite XAD-4 resin under rotatory stirring (1,200 rpm) at room temperature (23°C) for 40 min (Scalise et al., 2018a). The composition of the initial reconstitution mixture was: 50 μL of the purified WT protein or mutants (5 μg protein), 5 μL of 0.3 M EDTA, 340 μL of the mixture containing 100 μL of 10% egg yolk phospholipids (w/v) in the form of sonicated liposomes (Tonazzi et al., 2015) and 240 μL of 5% C_12_E_8_ or CHEMS as specified in the figure legends, 10 mM L-glutamine (except were differently indicated in the figure legend), 20 mM Hepes Tris pH 7.0 in a final volume of 700 μL. All the operations were performed at room temperature.

### Transport Measurements

To remove the external compounds prior functional uptake experiments, 600 μL of proteoliposomes was passed through a Sephadex G-75 column (0.7 cm diameter × 15 cm height) pre-equilibrated with 20 mM Hepes Tris pH 7.0 and sucrose at an appropriate concentration to balance the internal osmolarity. Uptake experiment was started by adding 50 μM [^3^H]glutamine and 50 mM Na-gluconate to 100 μL proteoliposomes, at 25°C. Transport reaction was stopped by adding 100 μM HgCl_2_; according to the inhibitor stop method, the same inhibitor was added at time zero to control samples (blanks) (Palmieri and Klingenberg, 1979). At the end of the transport, 100 μL of proteoliposomes was passed through a Sephadex G-75 column (0.6 cm diameter × 8 cm height) to separate the external from the internal liposomal radioactivity. Then, proteoliposomes were eluted with 1 mL 50 mM NaCl and collected in 3 mL of scintillation mixture, vortexed and counted. The experimental values were analyzed by subtracting to each sample the respective control (blank); the initial rate of transport was measured by stopping the reaction after 15 min, i.e., within the initial linear range of [^3^H]glutamine uptake into the proteoliposomes. Grafit 5.0.13 software was used to calculate kinetic parameters, to derive percent of residual activity values in inhibition assays and to measure transport rate by first-order rate equation.

### Cell Culture, Methyl-β-Cyclodextrin Treatment, and Transport Assay

HeLa cells were maintained in Dulbecco's Modified Eagle Medium (DMEM) supplemented with 10% (v/v) Fetal Bovine Serum (FBS), 1 mM glutamine and 1 mM sodium pyruvate under standard conditions, i.e., 37°C in a humidified incubator and a 5% CO_2_ atmosphere. Prior of methyl-β-cyclodextrin experiments, cells were plated on 12-well plates and treatment was performed when cells reached 70% confluence. Treatment with 10 mM methyl-β-cyclodextrin was performed for 60 min in the incubator in serum free medium (Dickens et al., 2017) and then, transport assay was performed as previously described (Console et al., 2015). In brief, cells were rinsed twice with warm transport buffer prepared with 20 mM TrisHCl pH 7.4, 10 mM BCH, and 10 mM MeAIB. Radiolabeled 10 μM [^3^H]glutamine was added together with 100 mM NaCl and the transport reaction was terminated after 60 s by discarding the uptake buffer and rinsing the cells three times with the same ice-cold transport buffer (500 μL per well per rinse). Cells from each well were solubilized in 500 μL of 1% TX-100 solution. Cell extracts were counted for radioactivity (400 μL). The remaining 100 μL in each well were used for protein concentration assay. Na^+^-dependent glutamine transport was evaluated by subtracting the transport values from those deriving from transport conducted in the absence of Na^+^.

### Cross-Link Reaction of hASCT2

The HeLa cells were plated on 10 cm^2^ dishes an treatment was performed when cells reached 70% confluence. Then, cells were treated with 0.75% formaldehyde for 10 min shaking at room temperature; the reaction was stopped using 125 mM glycine prepared in PBS for 5 min shaking at room temperature. Then, cells were collected and washed with PBS. Cells pellet were stored at −20°C. The purified WT hASCT2 protein, incubated in the absence or the presence of 0.75 mg CHEMS prepared as above described, was used for the cross-link reaction with formaldehyde diluted in 20 mM Tris HCl pH 9.0. The protein in both conditions was treated with increasing concentrations of the cross-linker for 2 min at 23°C, as detailed in the figure legends. The reaction was stopped adding cold 1.25 M glycine solubilized in PBS. Samples were prepared using loading dye with 10% SDS and heated for 5 min at 65°C. Samples were analyzed by SDS—PAGE followed by western blotting as described below.

### Internal Volume Measurement

The internal volume of proteoliposomes prepared with different amount of cholesterol was calculated using the colorimetric phosphate method as previously described (Indiveri et al., 1994). In brief, different proteoliposome samples were prepared including, in the reconstitution mixture, 50 mM dipotassium phosphate (K_2_HPO_4_). After elution from Sephadex G75, buffered without phosphate, 100 μL of sample were used for the colorimetric reaction with 150 μL of 10% SDS and 700 μL of solution R (prepared with 10 mM hexammonium heptamolybdate 4-hydrate, 0.3 mM of H_2_SO_4_, and 0.1 mM FeSO_4_). After incubating samples in the dark for 30 min, the absorbance was measured using spectrophotometer analysis (wavelength = 578 nm). Internal volume in μL was derived from nmol of phosphate included in liposomes.

### Molecular Docking Approach

To identify cholesterol binding sites in ASCT2 transporter (PDB: 6GCT), molecular docking was performed using Autodock 4.2 (Forli et al., 2016). At first, a blind docking was made, generating a grid which covered the whole trimer. The size of the grid box was set to 114 × 114 × 114 Å (x, y, and z). The optimized ligand molecule was docked into refined ASCT2. The best conformation space of the ligand was searched employing the Lamarckian Genetic Algorithm. Default parameters were used and 20 different conformers were generated for cholesterol molecule. Other six refined docking simulations were then carried out for accurate results. For each docking, a grid box with reduced size and space between gridpoints was generated in a specific area. For each docking process, the number of generation was reduced to 10 conformers. The best final pose was chosen giving priority to the lowest binding-energy conformation. As a further docking proof, a blind docking calculation was also performed with Achilles Blind Docking Server (https://bio-hpc.ucam.edu/achilles/) (Sanchez-Linares et al., 2012). Molecular graphics and visualization of the amino acids involved in the interaction with cholesterol were performed with the UCSF Chimera 1.13.1 software (Pettersen et al., 2004) (Resource for Biocomputing, Visualization, and Informatics, University of California, San Francisco, CA, USA).

### Other Methods

The amount of purified recombinant hASCT2 WT and mutants was estimated from Coomassie blue-stained 12% SDS–PAGE gels by using the Chemidoc imaging system equipped with Quantity One software (Bio-Rad) as previously described (Torchetti et al., 2011). The cross-linked samples were analyzed on 8% SDS-PAGE. For Western Blot analysis, hASCT2 was immuno-detected incubating membrane with conjugated anti-His antibody 1:10,000 in 3% BSA for 1 h at room temperature or with anti-hASCT2 (1:1,000) incubated overnight in 3% BSA under shaking at 4°C and then 1 h at room temperature with secondary antibody anti-rabbi (1: 5,000) in 1% BSA. The reaction was detected by Electro Chemi Luminescence (ECL) assay in the darkroom.

## Results

### Effect of Cholesterol on the hASCT2 Reconstitution and Function

**Figure 1:**
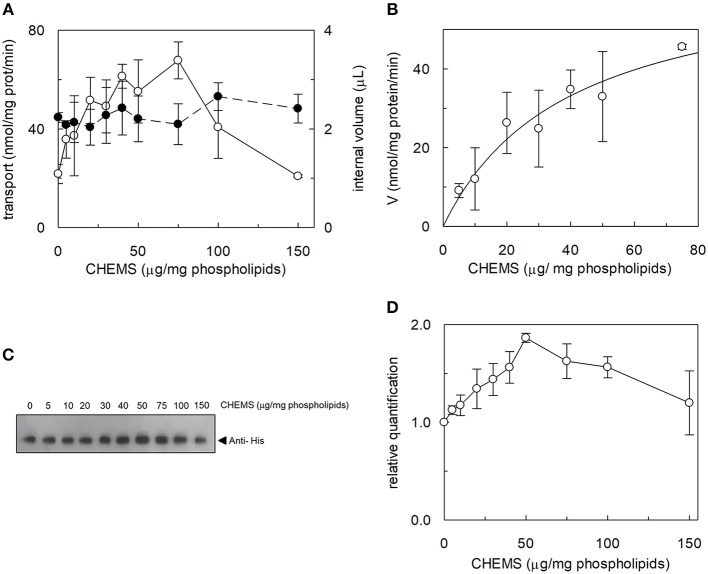
Effect of cholesterol on hASCT2 reconstitution in proteoliposomes. **(A)** Purified WT hASCT2 was reconstituted in proteoliposomes prepared with the indicated amounts of CHEMS per mg phospholipids as described in Materials and Methods. Transport assay was started adding 50 μM of [^3^H]glutamine and 50 mM Na-Gluconate to proteoliposomes containing 10 mM glutamine. Transport was measured in 20 min, i.e., within the initial linear part of the time course. Transport rate was expressed as nmol/mg prot/min (∘, left y-axis). In the right y-axis, the internal volume of proteoliposomes used for the transport assay was calculated as described in Materials and Methods (•). **(B)** Experimental data obtained from experiments shown in **(A)** were analyzed subtracting each value corresponding to a specific CHEMS amount to the condition in which CHEMS was not present in the reconstitution mixture. Data were then plotted using non-linear Michaelis- Menten equation as described in Materials and Methods excluding data of high CHEMS amount, i.e., 100 and 150 μg/mg phospholipids, because out of the plot range. **(C)** Samples (a volume corresponding to 1% of the total reconstitution mixture) derived from **(A)** were subjected to SDS-PAGE and western blotting analysis, as described in Materials and Methods, for evaluating the incorporation of hASCT2 into proteoliposomes prepared with the indicated amount of CHEMS. Recombinant protein, harboring a 6His tag at the C-terminus, was detected using anti-His antibody. **(D)** Relative quantification of band intensity from western blotting of **(C)**. In **(A,B)** data are means ± SD of three independent experiments. In **(C)**, a representative image of three different experiments; in **(D)** data are means ± SD of three different western blotting analysis.

**Figure 2:**
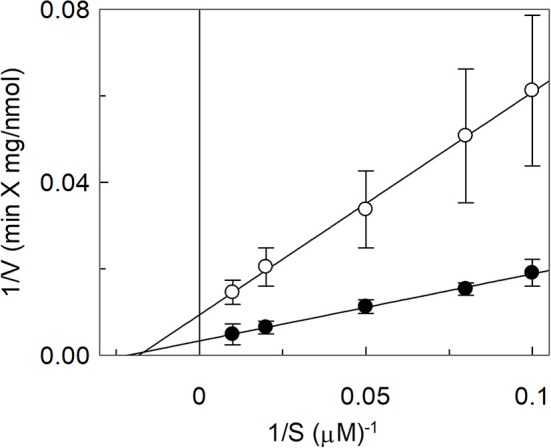
Effect of cholesterol on kinetics of hASCT2 reconstituted in proteoliposomes. Purified hASCT2 was reconstituted in proteoliposomes prepared without CHEMS (∘) or with 75 μg/mg phospholipids CHEMS (•) as described in Materials and Methods. Transport was started by adding indicated concentrations of [^3^H]glutamine and 50 mM Na-Gluconate to proteoliposomes containing 10 mM glutamine. Transport was stopped after 15 min and analysis of the data was performed according to linear Lineweaver–Burk plot as reciprocal transport rate vs. reciprocal glutamine concentration. Data are means ± SD of three independent experiments.

**Figure 3:**
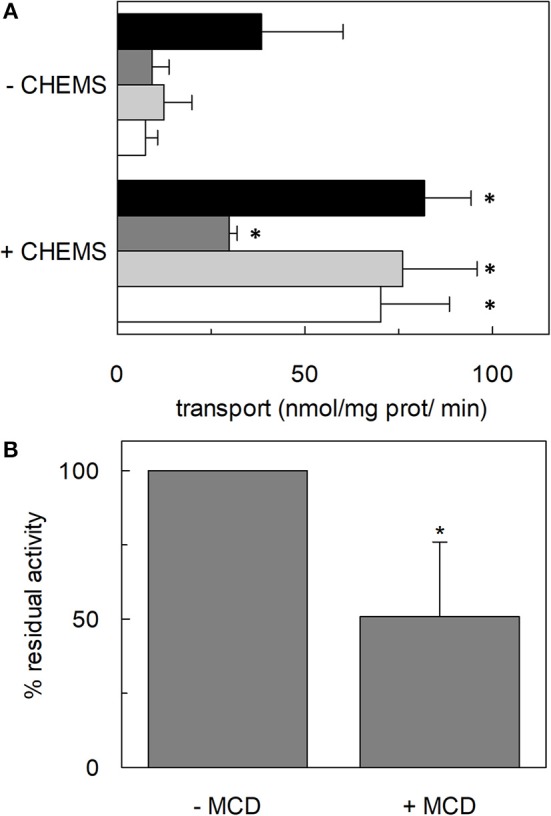
Effect of cholesterol on transport activity mediated by hASCT2. In **(A)**, purified hASCT2 was reconstituted in proteoliposomes prepared without CHEMS or with 75 μg/mg phospholipids CHEMS as indicated and described in Materials and Methods. Transport assay was started adding 50 μM of [^3^H]glutamine and 50 mM Na-Gluconate to proteoliposomes containing 10 mM glutamine (black bar) or asparagine (dark gray bar) or threonine (light gray bar) or serine (white bar). Initial transport rates were calculated as k x limit (nmol/mg prot/min) from the first-order rate equation used to plot the time course data. Data are means ± SD of three independent experiments. In **(B)**, HeLa cells were treated with 10 mM methyl-β-cyclodextrin (MCD) as described in Materials and Methods. Transport was started adding 10 μM [^3^H]glutamine in the presence or in the absence of 100 mM NaCl. Transport activity was calculated as the percent of residual activity with respect to the control condition (without MCD in the transport assay). ^*^Significantly different from the control sample (proteoliposomes prepared without CHEMS in **A** or without MCD in **B**) as estimated by Student's t-test (*P* < 0.05).

The effect of cholesterol on the transport function of ASCT2 was studied using the proteoliposome experimental model which allows modifying the lipid composition of the membrane. Cholesterol, in the form of cholesteryl hemisuccinate (CHEMS), was added to the lipid/detergent mixture before the formation of proteoliposomes, as described in Materials and Methods. As shown in Figure 1A, cholesterol strongly stimulated the transport activity of hASCT2 measured as sodium-dependent glutamine antiport (${\text{Na}}_{\text{ex}}^{+}$-[^3^H]glutamine_ex_/glutamine_in_). Maximal stimulation was observed at a cholesterol concentration of 75 μg/mg total lipids corresponding to 7.5% cholesterol which falls within the physiological cholesterol concentration in cells (Litvinov et al., 2018); at higher concentrations, the activity dramatically decreased. To investigate whether the increase of [^3^H]glutamine accumulation could simply be due to an increase in the internal space of proteoliposomes caused by the inclusion of cholesterol, the internal volume was measured. Nearly no variations were observed at the various concentration of cholesterol, with respect to the control, indicating that cholesterol does not affect the internal proteoliposome volume (Figure 1A). Thus, the stimulation may be caused by an effect of cholesterol in improving the protein insertion into the membrane and/or the kinetics. The hyperbolic trend of the data up to 75 μg/mg total lipids (Figure 1B) observable after subtracting the control value (absence of cholesterol), correlates with a saturation process which is typical of protein-ligand interactions. A further increase in cholesterol concentration impaired the transport function. To gain further insights into the mechanism of stimulation of the transport activity, the amount of protein incorporated into proteoliposomes at increasing cholesterol concentrations was detected (Figure 1C). As shown by the Western blot, protein incorporation increased by increasing the cholesterol concentration with a maximum at 50 μg/mg total lipids of cholesterol. Thus, the increase in reconstituted protein amount did not exactly follow the increase in transport activity (Figure 1D), as indicated by the low correlation coefficient of 0.78 calculated for the two sets of data. Taken together, these results showed that the effect on reconstitution of hASCT2 into proteoliposomes is not the sole responsible for transport stimulation. To gain further insights into these aspects, kinetics was studied in the absence or presence of cholesterol (Figure 2). The K_m_ for glutamine was not substantially changed in the absence or presence of cholesterol while the V_max_ increased about three times (113 ± 26.6 nmol **·** mg ^−1^ **·** min^−1^ and 370 ± 79.3 nmol **·** mg ^−1^ **·** min^−1^ in the absence or the presence of cholesterol, respectively). Furthermore, to evaluate the effect of cholesterol in the transport cycles resembling those occurring in physiological conditions, the heterologous exchanges Na^+^-[^3^H]glutamine_ex_/asparagine_in_, Na^+^-[^3^H]glutamine_ex_/threonine_in_, or Na^+^-[^3^H]glutamine_ex_/serine_in_, were measured (Figure 3A). Interestingly, the addition of cholesterol was much more effective on the heterologous antiport of [^3^H]glutamine_ex_/serine_in_ and [^3^H]glutamine_ex_/threonine_in_ (six and nine times stimulation, respectively) than on the homologous antiport (two times stimulation) or the heterologous [^3^H]glutamine_ex_/asparagine_in_ antiport (three times stimulation). The effect of cholesterol was shown also in intact HeLa cells treated with methyl-β-cyclodextrin, which is a known cholesterol depleting reagent (Dickens et al., 2017). In line with results obtained in proteoliposomes the Na^+^-dependent glutamine transport was impaired upon cholesterol deprivation (Figure 3B).

### Docking of Cholesterol to the 3D Structure of hASCT2

**Figure 4:**
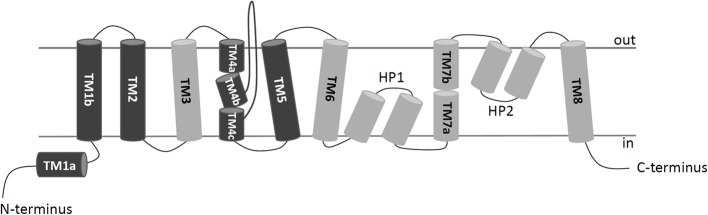
2D schematic representation of a protomer organization. TransMembrane domains (TMs) of hASCT2 are depicted in dark gray (scaffold domain) or light gray (transport domain); HP1 and HP2 hairpins connect TM6-8; loops between TMs are indicated in lines. N- and C-termini of the protein face to the intracellular space delimited by the membrane.

**Figure 5:**
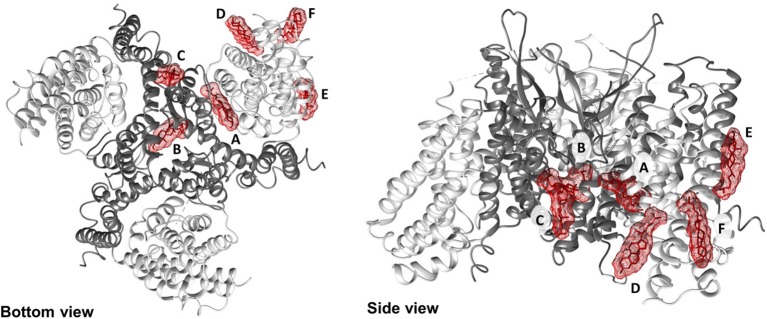
Docking of cholesterol molecules with hASCT2. Bottom view (left) and side view (right) of the trimeric hASCT2 (PDB: 6GCT). Cholesterol molecules, indicated by letters A, B, C, D, E, and F are depicted as red sticks with mesh surface interacting with the scaffold domain (TMs 1,2,4,5 in dark gray ribbon) and with the transport domain (TMs 3,6,7,8, HP1 and HP2 in the light gray ribbon) of a protomer. The numbering of TMs corresponds to those described in Figure 4. Dotted gray lines indicate missing residues in the 3D structure (PDB: 6GCT). The structure was visualized using Chimera 1.13.1 software.

**Figure 6:**
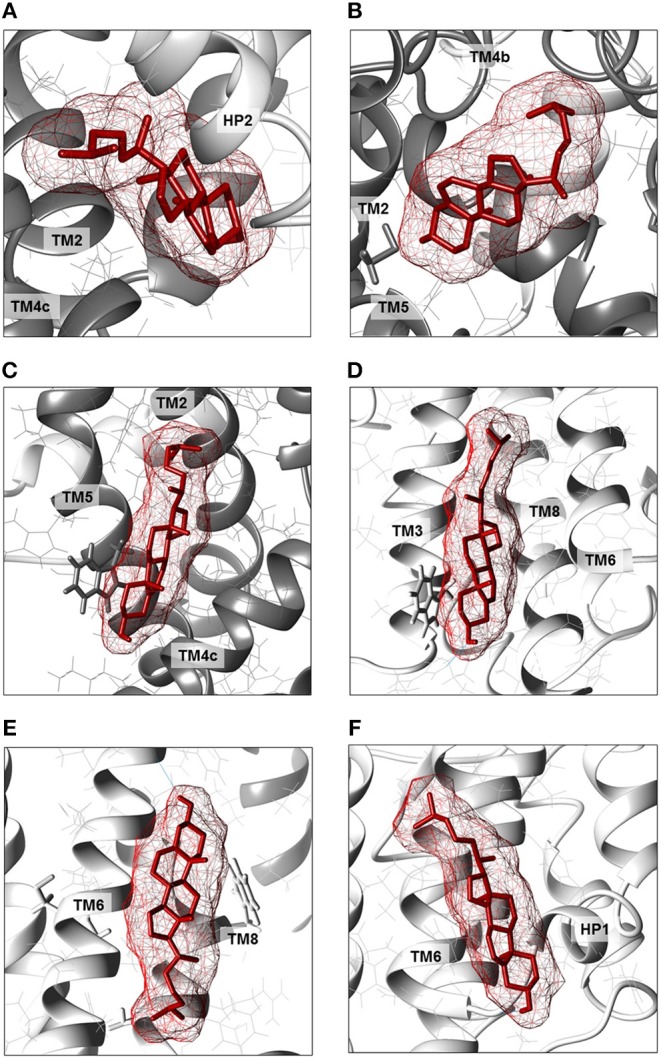
Enlargements of cholesterol poses. Cholesterol molecules are depicted as red sticks with mesh surface facing to residues of neighboring helices in scaffold domain (dark gray ribbon) or transport domain (light gray ribbon). **(A)** Cholesterol facing to TMs 2, 4c (scaffold domain) and HP2 (transport domain) of a protomer. **(B)** Cholesterol facing to TMs 2, 4b and 5 (scaffold domain) of two protomers; in gray stick, residue C110. **(C)** Cholesterol facing to TM4c (scaffold domain) of a protomer and TM2 and TM5 (scaffold domain) of the adjacent protomer; in gray stick, residue W272. **(D)** Cholesterol facing to TMs 3, 6 and 8 (transport domain) of a protomer; in gray stick, residue W130. **(E)** Cholesterol facing to TM6 (transport domain) including CARC motif and TM8 (transport domain); in gray stick, residues C308 and C309 (TM6) and W461 and C467 (TM8). **(F)** Cholesterol facing to TM6 (transport domain) including CRAC motif and HP1 (transport domain) of a protomer. Except those indicated in gray stick, all residues are indicated as gray wire. The structure was visualized using Chimera 1.13.1 software.

To shed new lights on the molecular mechanisms underlying effects of cholesterol on the transport activity of ASCT2 and in agreement with the data of Figure 1, direct binding of cholesterol to the protein was hypothesized. This correlated well with previous structural data that showed electron density areas in the 3D structure of the hASCT2 trimer (Garaeva et al., 2018; Yu et al., 2019). To predict the possible sites of interaction, docking of cholesterol into the hASCT2 trimer was performed. As shown in Figures 4–6, at least six sites were predicted. Some of these poses, i.e., those indicated by A, B, C, and D well overlap the electron density areas in the recently published 3D structures (Garaeva et al., 2018; Yu et al., 2019). Interestingly, two additional poses, indicated by the letters E and F, resulted from the docking. Noteworthy, the mentioned poses E and F were docked close to CARC (pose E, TM6) and CRAC (pose F, TM6) motifs that are well-acknowledged binding sites for cholesterol. CRAC is an acronym standing for Cholesterol Recognition/interaction Amino acid Consensus sequence, while CARC is considered as an inverted CRAC domain (Fantini and Barrantes, 2013; Fantini et al., 2019). Moreover, pose D (TM3), also present in the 3D structure, is docked close to a R-W-L domain that is considered a simil-CARC/CRAC domain (Fantini and Barrantes, 2013). To prove that indeed cholesterol binds to the protein, a strategy based on the specific targeting of residues located in the neighborhood of cholesterol molecules was employed. According to this approach, we sought to evaluate the possible prevention of chemical targeting by the cholesterol added to the proteoliposomes.

### Targeting Tryptophan Residues by the Koshland's Reagent

**Figure 7:**
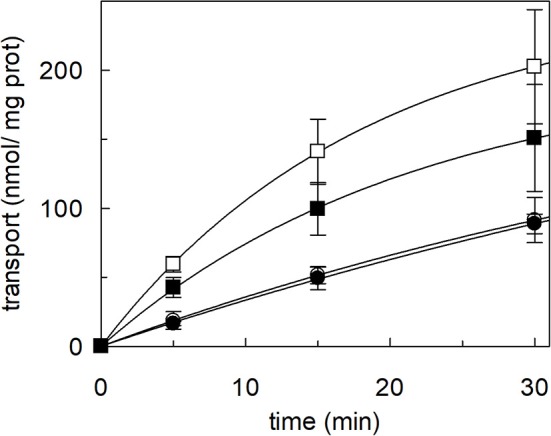
Effect of Koshland's reagent on cholesterol stimulation of hASCT2 reconstituted in proteoliposomes. Purified hASCT2 was incubated (•, ■) or not (∘, □) with 0.5 mM Koshland's reagent prior reconstitution. After treatment with the Koshland's reagent, the protein was reconstituted in proteoliposomes prepared without CHEMS (∘, •) or with 75 μg/mg phospholipids CHEMS (□, ■) as described in Materials and Methods. Transport assay was started adding 50 μM of [^3^H]glutamine and 50 mM Na-Gluconate to proteoliposomes containing 10 mM glutamine. Transport was stopped as described in Materials and Methods at the indicated time points. Values were plotted according to the first-order rate equation. Data are means ± SD of three independent experiments.

The Koshland's reagent was employed due to its acknowledged specific reactivity toward tryptophan residues at pH 7.0. In particular, the reaction triggers a chemical modification of the indole ring of the tryptophan side chain (Loudon and Koshland, 1970). The purified WT hASCT2 was treated with Koshland's reagent as described in Materials and Methods in the presence or the absence of cholesterol (Figure 7). The treatment of the protein with the reagent had nearly no effect in the absence of cholesterol indicating that the modification of such residues does not influence the protein activity, i.e., tryptophan residues are not crucial for transport function. The reagent, on the contrary, prevented the stimulation exerted by cholesterol by roughly 30%, indicating that tryptophan residues might be critical for the interaction with cholesterol thus confirming the predicted location of cholesterol in the vicinity of those residues (poses letters C, D, and E; see related Figure 6).

### Targeting Cysteine Residues With SH-Reagents

**Figure 8:**
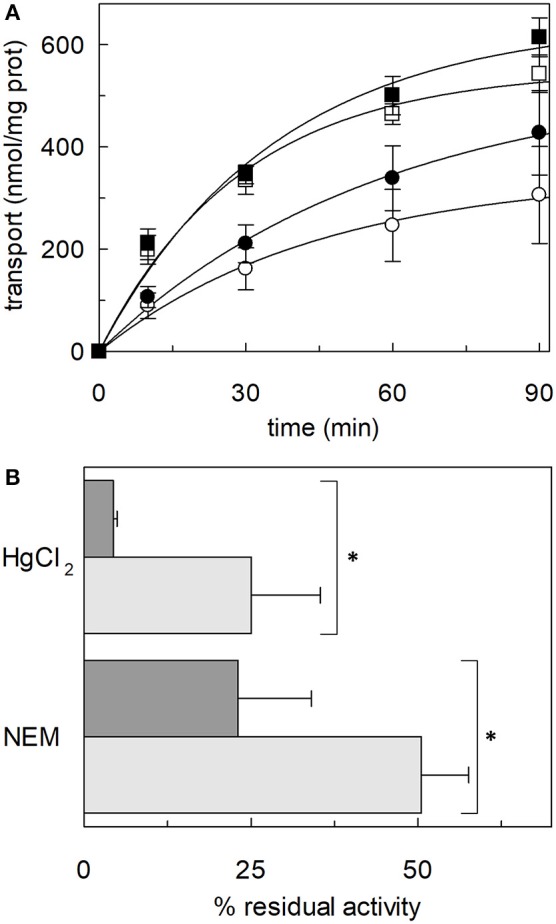
Effect of SH reagents on the transport activity of hASCT2 reconstituted in proteoliposomes. **(A)** Effect of DTE. Purified hASCT2 was reconstituted in proteoliposomes prepared without CHEMS (∘, •) or with 75 μg/mg phospholipids CHEMS (□, ■) as described in Materials and Methods. Transport was started by adding 50 μM [^3^H]glutamine and 50 mM Na-Gluconate in the absence (∘, □) or the presence (∙, ■) of 10 mM DTE, to proteoliposomes containing 10 mM glutamine. Transport was stopped as described in Materials and Methods at the indicated time points. Values were plotted according to the first-order rate equation. **(B)** Effect of HgCl_2_ and NEM. Purified hASCT2 was reconstituted in proteoliposomes prepared without CHEMS (dark gray bars) or with 75 μg/mg phospholipids CHEMS (light gray bars) as described in Materials and Methods. Transport was started by adding 50 μM [^3^H]glutamine and 50 mM Na-Gluconate, in the presence of 20 μM HgCl_2_ or 0.5 mM NEM, to proteoliposomes containing 10 mM glutamine. Transport was measured in 20 min. Transport activity was calculated as the percent of residual activity with respect to the control condition (without any addition in the transport assay). ^*^Significantly different from the control sample (proteoliposomes prepared without CHEMS) as estimated by Student's *t*-test (*P* < 0.05). In **(A,B)** data are means ± SD of three independent experiments.

**Figure 9:**
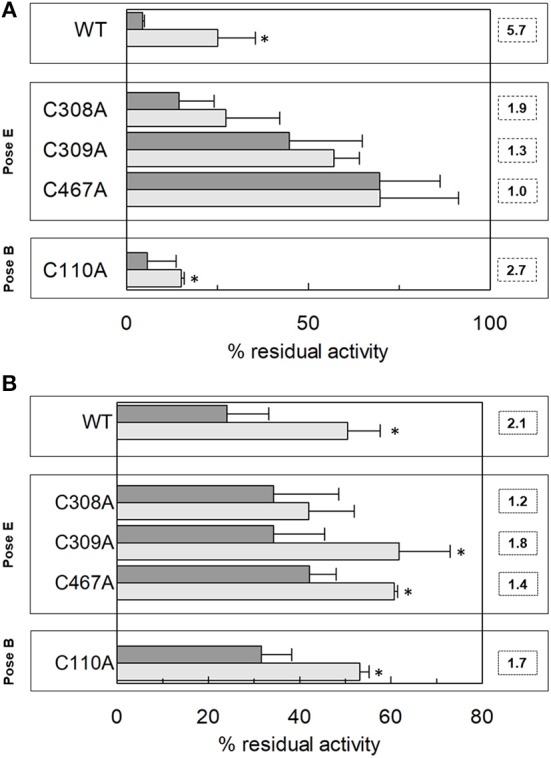
Effect of HgCl_2_ and NEM on transport activity of hASCT2 WT and Cys mutants reconstituted in proteoliposomes. Effect of HgCl_2_ **(A)** or NEM **(B)**. Purified hASCT2 WT, C308A, C309, C467A or C110A was reconstituted in proteoliposomes prepared without CHEMS (dark gray bars) or with 75 μg/mg phospholipids CHEMS (light gray bars) as described in Materials and Methods. Transport was started by adding 50 μM [^3^H]glutamine and 50 mM Na-Gluconate in the presence of 20 μM HgCl_2_ **(A)** or 0.5 mM NEM **(B)**, to proteoliposomes containing 10 mM glutamine. Transport was measured in 20 min. The boxes grouped the residues corresponding to the indicated poses (see Figure 6). Dotted boxes indicated the protection index calculated as the ratio between residual activity in the presence of CHEMS and the residual activity in the absence of CHEMS. Transport activity was calculated as the percent of residual activity with respect to the control condition (without any addition in the transport assay). ^*^Significantly different from the control sample (proteoliposomes prepared without CHEMS) as estimated by Student's *t*-test (*P* < 0.05). Data are means ± SD of three independent experiments.

Following the same experimental setup, changes of the reactivity toward SH reagents was also investigated considering that the poses B and E are predicted to be in the vicinity of cysteine residues. Previous work showed that hASCT2 transport activity is stimulated by DTE that reduces thiol residues of cysteines (Scalise et al., 2018b); interestingly, in the presence of cholesterol, the stimulation by DTE was nearly abolished (Figure 8A). Interestingly, the addition of DTE had no effect on transport measured in HeLa cells (not shown) where the ASCT2 should have an optimal cholesterol milieu. To further investigate this issue, the transporter was treated with SH reagents previously shown to interact with the protein, causing transport inhibition. The treatment of WT hASCT2 with HgCl_2_ was performed using proteoliposomes prepared in the presence or absence of cholesterol; as expected, HgCl_2_ exerted strong transport inhibition (Figure 8B); interestingly, the presence of cholesterol protected from the inhibition. A similar experiment was performed with the SH alkylating reagent NEM and, also, in this case, significant protection by cholesterol was observed (Figure 8B). These results indicated that some cysteine residues of the hASCT2 are masked by one or more bound cholesterol molecules (Figures 6B,E). The availability in our laboratory of some cysteine mutants (Scalise et al., 2018b), together with the previous finding that C467 is the major target of SH reagents, allowed us to map the presence of the new predicted cholesterol pose E (Figure 6E) facing to the C467 residue. Therefore, the extent of inhibition by HgCl_2_ was evaluated on three mutants, i.e., C308A, C309A, and C467A reconstituted in proteoliposomes prepared in the absence or in the presence of cholesterol (Figure 9A). The data showed that the protection of HgCl_2_ binding and inhibition by cholesterol in the mutants was different with respect to the WT. The protection index was calculated as the ratio between residual activity in the presence and in the absence of cholesterol (dotted boxes in the Figure 9A) confirming that protection observed in the WT was lost or much smoothed in the three mutants indicating that a cholesterol molecule may mask one or more of the three cysteine residues. Furthermore, the same experiment was conducted using the C110A mutant (Figure 9A) since this residue lies in the neighborhood of the cholesterol pose B which corresponded to an electron density area in the previously published 3D structure (Figure 6B). Also, in this case, protection was observed. However, differently from C308A, C309A, and C467A data, the protection index was more similar to that of the WT. This may indicate that either the cysteine is targeted by HgCl_2_ without consequences for the transport activity, or that this cysteine residue is not easily accessible to the provided reagent given its location in the core of the scaffold domain of the homotrimer (Figure 4). Therefore, the alkylating reagent NEM was employed on the C308A, C309A, C467A, and C110A mutants (Figure 9B). Interestingly, the observed results were very similar to those obtained with HgCl_2_ even if the extent of inhibition and, hence, of protection by cholesterol were less strong than those observed using HgCl_2_.

### Effect of Cholesterol on the Homotrimer Formation of hASCT2

**Figure 10:**
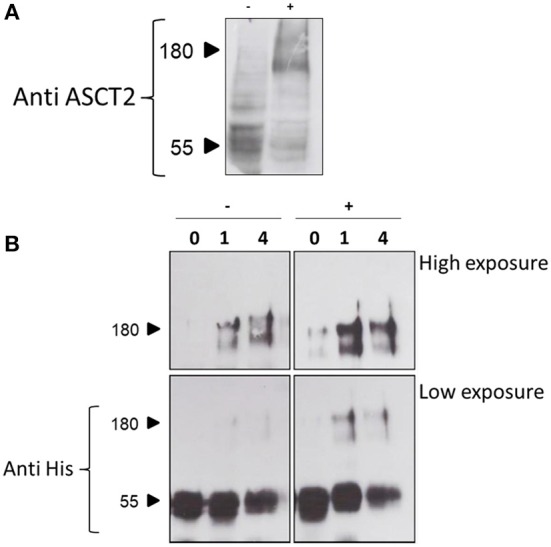
Cross-linking analysis of hASCT2. In **(A)**, immunoblot analysis of protein extracted from HeLa cells treated with 0.75% formaldehyde as described in Materials and Methods. Samples (50 μg total lysate) were subjected to SDS-PAGE and immunoblot with anti-hASCT2 (1:1,000); (–), control without formaldehyde; (+), sample treated with formaldehyde. In **(B)**, immunoblot analysis of purified hASCT2 treated with 1 or 4% formaldehyde as described in Materials and Methods. Samples were subjected to SDS-PAGE and immunoblot with anti-hASCT2 (1:1,000).

The 3D structure of ASCT2, as well as the previous homology models obtained on the GltPh and on the hEAAT1 (Yernool et al., 2004; Canul-Tec et al., 2017), share a trimeric organization (Garaeva et al., 2018; Scalise et al., 2018b; Yu et al., 2019). This oligomeric form is very probably the functional one as it was indicated by previous kinetic and functional studies (Pingitore et al., 2013; Scalise et al., 2014). To address this issue, a cross-linking approach was employed in intact cells using formaldehyde (Sutherland et al., 2008). The experiment showed that in HeLa cells ASCT2 is mostly assembled in a trimeric form (Figure 10A). The effect of cholesterol on the trimeric form of hASCT2 was further evaluated using the recombinant purified protein in a cross-linking reaction conducted by adding cholesterol to the protein. The western blot analysis suggested that the presence of cholesterol facilitated the formation of ASCT2 trimer with respect to the samples with no added cholesterol (Figure 10B) as indicated by both the decrease of the monomer band and the increase of the band at a triplicate apparent molecular mass.

## Discussion

### Interaction Between Cholesterol and Membrane Proteins

In the recent years, the studies on SLCs received more and more attention for either physiological and pathological aspects given their well-acknowledged role in mediating traffic of nutrients, catabolites, drugs, and xenobiotics across cell membranes and within cells across intracellular compartments. Besides function and kinetics, characterization of regulatory properties of membrane transporters represents a novel and promising field of investigation that is still *in nuce* (Cesar-Razquin et al., 2015). In this scenario, a very important issue is the influence that the lipid milieu can exert on transporter function. Among membrane lipids, cholesterol is of special interest since besides influencing physical properties of membranes (Yang et al., 2016; Fantini et al., 2019), it mediates the interaction between the transmembrane domains of proteins and the membrane interior thereby modulating protein function (Fantini and Barrantes, 2013). Noteworthy, over the years, some protein motifs have been described which are responsible for cholesterol binding: the best known are the CRAC and CARC (inverted CRAC) motifs (Li and Papadopoulos, 1998). Binding of cholesterol to transmembrane domains may also occur at motifs not completely overlapping CARC and CRAC (Fantini and Barrantes, 2013). This scenario has been depicted for different eukaryotic membrane transporters; interestingly, the first report dealing with cholesterol effects on a eukaryotic membrane transporter has been published in the 1970s. In this work, it has been shown that the activity and also the affinity of a glucose transporter is modulated by sterols (Komor et al., 1974). Later on, the effect of cholesterol has been also evaluated on the Na^+^ pump in human blood cells (Giraud et al., 1976) and also on ion channels (Brini et al., 2017). Recently, other studies have been conducted and more refined results have been obtained thanks to the resolution of 3D structures such as in the case of DAT from *Drosophila melanogaster* (Penmatsa et al., 2013), hSERT (Coleman et al., 2016), hLAT1 (Yan et al., 2019).

### Combination of Computational Analysis and Chemical Targeting Approaches

In the current work, a combined strategy of experimental and computational approaches has been used for describing the effects of cholesterol bound to the hASCT2. It is worth noting that docking of cholesterol is particularly challenged by its hydrophobic nature and, hence, experimental validation is useful to confirm the predictions (Listowski et al., 2015). The employed strategy allowed us to compare the transport function and kinetics under conditions of absence (or low content) with the increased cholesterol content. The range of used cholesterol concentrations does not affect the size of proteoliposomes and, hence, the observed effects are due to direct interaction with the protein. The presence of saturable sites for cholesterol on hASCT2 has been suggested by the hyperbolic dependence of transport activity on cholesterol concentrations. This has been further confirmed by the activity impairment at higher cholesterol concentrations (Figure 1). The results correlate with the presence of multiple cholesterol sites on each protein monomer as observed in the case of the nicotinic receptor (Baier et al., 2011). Interestingly, cholesterol did not change the K_m_ for glutamine, while strongly affected the initial transport rate (Figures 2, 3). This finding indicates that the structure and/or the binding properties of the active site do not vary, while cholesterol probably influences the rate of conformational changes that are at the basis of the elevator mechanism of transport. Indeed, four out of six poses are on the transport domain: three cholesterol molecules face toward the lipid bilayer, one faces toward the scaffold domain. Cholesterol may play a major role in mediating the interaction with the phospholipid bilayer favoring the sliding of the elevator. This finding can be relevant in terms of the biological function of ASCT2 favoring an increase of glutamine uptake when cells undergo high proliferation (Scalise et al., 2018a). In this condition, glutamine is exchanged with smaller amino acids such as serine allowing the net uptake of at least one carbon atom used in the truncated form of TCA for energy production from glutamine carbon skeleton (Scalise et al., 2017b). Interestingly, the reaction serine_in_/glutamine_ex_ was stimulated by cholesteryl hemisuccinate more than the other hetero-exchanges (Figure 3A). This indicates that cholesterol may exert a specific modulation of serine binding to the internal site, increasing the rate of conformational changes underlying the transport reaction. Indeed, allosteric sites for lipid binding have been recently described in the ASCT2 structure (Garaeva et al., 2019). Therefore, it can be speculated that cholesterol may act as another key regulation point of hASCT2 transport activity and/or stability in the plasma membrane, required in both physiological and pathological conditions. Interestingly, cholesterol membrane content is modulated in cancer cells (Garcia-Bermudez et al., 2019). It is worth noting that docking performed with cholesteryl hemisuccinate or cholesterol perfectly overlapped (not shown) in line with the observation that cholesteryl hemisuccinate and cholesterol show similar properties in cell membranes and the interaction with proteins. Indeed, cholesteryl hemisuccinate is virtually always used for studying protein-cholesterol interaction (Kulig et al., 2015).

### Identification of Cholesterol Poses on ASCT2 Transport Protein

Interestingly, the pose shown in Figure 6C occupies a site which overlaps the allosteric site described in SLC1A3 as the binding site of the inhibitor UCPH_101_ (Canul-Tec et al., 2017). In addition, the pose shown in Figure 6D is included in a non-canonical cholesterol binding motif very close to another key regulatory point of hASCT2, that is the PDZ binding domain; interestingly, cholesterol has been shown to regulate also the interactions between PDZ binding domain and scaffold proteins (Sheng et al., 2012). Furthermore, the protection by cholesterol on the SH reagents HgCl_2_ and NEM is prevented in the case of cysteine mutants C308 and C309 which lie on the CARC motif of the pose E. When looking at the C467A mutant the protection by cholesterol was not measurable. This may indicate that in the WT protein, cholesterol that binds in the vicinity of C308 and C309 residues, can reduce the inhibition by HgCl_2_ caused by binding of the reagent to C467. When this cysteine residue is missing, also protection by cholesterol is less evident. It is important to note that C467 is one of the residues responsible for substrate recognition in hASCT2 but cholesterol does not affect the K_m_ for glutamine. Indeed cholesterol does not enter the substrate-binding site (pose E) but is peripherally located and exposed toward the residues C308, C309. This location, therefore, only influences the environment involved in the reagent reaction with C467. Cholesterol molecules are also predicted and described in the 3D structure (Yu et al., 2019) to interact with the scaffold domain. Thus, besides the effect on the function of hASCT2, cholesterol may play also a role in the stabilization of the trimer. Indeed, the two poses interacting with the scaffold domain (Figures 6B,C), take contact with two different subunits. The cross-linking experiment on the recombinant protein confirms the hypothesis. The trimer formation is, indeed, required for the proper functionality of the protein which is almost exclusively present in a trimeric form in the plasma membrane (Figure 10A). In conclusion, the suggested physical interaction of cholesterol with hASCT2 has been confirmed at the functional level by employing biochemical and bioinformatics approach. Further work is in the course to better define the molecular determinants of such interactions and to identify other potential sites.

## Data Availability Statement

The datasets generated for this study are available on request to the corresponding author.

## Author Contributions

MS and CI conceived, designed the experiments, and analyzed the data. MS and JC performed proteoliposome functional assays. EA performed docking analysis. LP prepared yeast constructs and optimized yeast cell growth. TM, AE, and LC performed yeast cell growth for protein over-expression and purification. MS, JC, and CI wrote the paper. CI supervised the work.

### Conflict of Interest

The authors declare that the research was conducted in the absence of any commercial or financial relationships that could be construed as a potential conflict of interest.

## Abbreviations

SLC: SoLute Carrier
GSH: Reduced Glutathione
H_2_S Hydrogen Sulfide
NO: Nitric Oxide
mTOR: mammalian Target of Rapamicyn
CHEMS: Cholesteryl HemiSuccinate
β-MCD: Methyl-beta-CycloDextrin
Cryo-EM: Cryo Electron Microscopy
C_12_E_8_: Octaethylene glycol monododecyl ether
YPDS: Yeast Extract Peptone Dextrose Sorbitol
BMGY: Buffered Glycerol-complex Medium
BMMY: Buffered Methanol-complex Medium
DTE: DiThioErythritol
NEM: N-ethylmaleimide
BSA: Bovine Serum Albumin
CRAC: Cholesterol Recognition/interaction Amino acid Consensus sequence.
